# Malignant Portal Vein Thrombosis in Adenosquamous Carcinoma of the Gall Bladder

**DOI:** 10.7759/cureus.75790

**Published:** 2024-12-16

**Authors:** Vishnu Prasad Pulappadi, Krithika Rangarajan, Sunil Kumar

**Affiliations:** 1 Interventional Radiology, Kovai Medical Center and Hospital, Coimbatore, IND; 2 Radiology, All India Institute of Medical Sciences, New Delhi, New Delhi, IND; 3 Surgical Oncology, All India Institute of Medical Sciences, New Delhi, New Delhi, IND

**Keywords:** cancer, computed tomography, gall bladder, portal vein, thrombus

## Abstract

We report a rare case of adenosquamous carcinoma of the gall bladder (GB) causing portal vein tumor thrombus. A 40-year-old gentleman presented with acute-onset right upper abdominal pain. Ultrasonography revealed multiple calculi in the GB with wall thickening, suggesting acute cholecystitis. Intraoperatively, irregular wall thickening of GB with infiltration into the duodenum and stomach was found. Partial cholecystectomy was performed, and a histopathological examination of the resected specimen revealed adenosquamous carcinoma. PET-CT revealed a fluorodeoxyglucose (FDG)-avid lesion in the GB and the main portal vein, suggesting a tumor thrombus. Two weeks later, he developed jaundice, and contrast-enhanced CT (CECT) showed a heterogeneously enhancing mass in the GB fossa, encasing the main portal vein, with an enhancing filling defect within its lumen, suggestive of tumor thrombus. Multiple periportal and peripancreatic lymph nodes were seen, encasing the common bile duct and causing intrahepatic biliary radical dilation. Percutaneous transhepatic biliary drainage was performed, and palliative chemotherapy was started.

## Introduction

Carcinoma of the gall bladder (GB) is the most common malignancy of the biliary tract and is highly aggressive with an overall poor prognosis [1]. A large proportion of patients present in an advanced stage of the disease with non-specific symptoms. Only a small number of cases are diagnosed before surgery, and most of them are unresectable at the time of diagnosis [1]. Adenocarcinoma is the most common histological type of GB carcinoma and is followed in prevalence by squamous, adenosquamous, and papillary carcinomas [1]. It spreads through direct invasion, lymphatic and hematogenous routes, and peritoneal dissemination. Direct invasion is the predominant route of spread and commonly involves the liver, colon, duodenum, pancreas, and bile ducts.

Malignant venous thrombus occurs due to the contiguous extension of the tumor into the vein and is commonly seen in renal cell carcinoma, hepatocellular carcinoma, and adrenocortical carcinoma. Although hepatocellular carcinoma has a high propensity for invasion into the portal vein, only a few cases of malignant portal vein thrombus have been reported with GB carcinoma [2,3]. We report a case of adenosquamous carcinoma of GB presenting with a tumor thrombus in the portal vein.

## Case presentation

**Figure 1:**
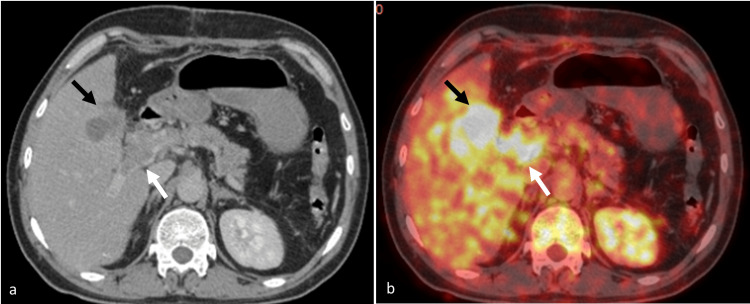
PET-CT showing the gall bladder mass and the tumor thrombus: (a) axial contrast-enhanced CT image shows heterogeneously enhancing irregular mass in the gall bladder fossa (black arrow), infiltrating segment 5 of the liver and surrounding fat stranding. An enhancing filling defect is seen in the main portal vein (white arrow), with expansion of the lumen, suggestive of tumor thrombus. (b) The corresponding PET-CT image shows uptake in the mass in the gall bladder fossa (black arrow) and the tumor thrombus (white arrow)

**Figure 2:**
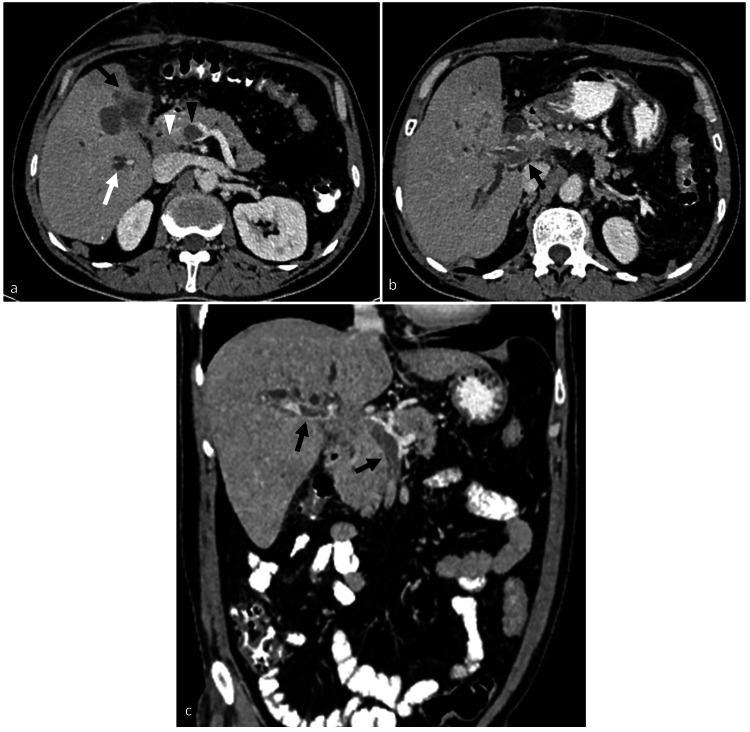
CT images showing the extension of tumor thrombus: (a) axial contrast-enhanced CT image obtained two weeks after the PET-CT shows the heterogeneously enhancing mass in the gall bladder fossa (black arrow), infiltrating the porta hepatis and adjacent liver parenchyma. Intrahepatic biliary radical dilation (white arrow), periportal lymphadenopathy (white arrowhead), and non-enhancing bland thrombus in the superior mesenteric vein (black arrowhead) are seen. (b) Axial contrast-enhanced CT image shows that the main portal vein is expanded with an enhancing filling defect within (arrow), suggesting tumor thrombus. (c) Coronal reformatted contrast-enhanced CT image shows a non-enhancing bland thrombus within the right portal vein and the superior mesenteric vein (arrows)

A 40-year-old gentleman, with no prior co-morbidities, initially presented to another center with acute-onset abdominal pain in the right upper quadrant. Ultrasonography of the abdomen was performed, which revealed multiple calculi in the GB with symmetrical thickening of the wall. A liver function test was done, and it was found to be normal. A clinical diagnosis of cholelithiasis with acute cholecystitis was made, and the patient subsequently underwent laparotomy one week later at another center. Intraoperatively, irregular wall thickening of the GB was seen, infiltrating the duodenum and the stomach. Also, the GB lumen was filled with pus, with greater omentum adhered to its fundus and body. Partial cholecystectomy was performed, and histopathological examination of the resected specimen revealed grade two moderately differentiated adenosquamous carcinoma with transmural invasion. The patient presented to our hospital one month after the surgery. A PET-CT was performed when the patient presented to our hospital, and it revealed a fluorodeoxyglucose (FDG)-avid lesion (maximum standardized uptake value (SUVmax)-3.97) in the GB with infiltration into the adjacent liver parenchyma. An enhancing filling defect was seen within the main portal vein showing avid FDG uptake (SUVmax-3.69), suggestive of a malignant thrombus (Figure 1). No focal lesion or intrahepatic biliary radical dilation was seen in the liver. No metabolically active lesions were seen in the rest of the body. The patient then developed jaundice, and his serum bilirubin increased from 1.5 to 10.5 mg/dL over 10 days. Contrast-enhanced CT (CECT) was performed to assess the cause of progressive jaundice, and it showed a heterogeneously enhancing mass in the GB fossa, infiltrating into segments 4B and 5 of the liver (Figure 2a). The mass encased the main portal vein, with an enhancing filling defect within its lumen, suggesting a tumor thrombus (Figure 2b). Non-enhancing bland thrombus was also seen within the right portal vein and the superior mesenteric vein and its tributaries with the formation of periportal collateral channels (Figure 2c). Multiple enlarged conglomerate lymph nodes were seen in the periportal and peripancreatic locations, encasing the common bile duct and causing bilobar intrahepatic biliary radical dilation, which was not seen in the PET-CT performed two weeks earlier. The mass was also seen to encase the common hepatic artery. No metastatic lesions were seen in the liver.

Left-sided percutaneous transhepatic biliary drainage was performed to relieve obstructive jaundice, following which the serum bilirubin reduced to 2 mg/dL. As there was extensive vascular encasement, definitive surgery was not possible. Palliative chemotherapy was therefore started for the patient. Two months following the initiation of palliative chemotherapy, the performance status of the patient deteriorated to score 4 on the Eastern Cooperative Oncology Group (ECOG) scale, and he was placed on best supportive care.

## Discussion

Although invasion into adjoining organs is common in carcinoma of GB, intraluminal invasion of the portal vein is rare. In our case, the tumor was seen encasing and infiltrating the main portal vein, resulting in tumor thrombus, as evidenced by enhancing filling defect within its lumen. A non-enhancing bland thrombus was seen in the right portal vein and the superior mesenteric vein, likely due to stasis of the blood flow. Portal vein tumor thrombus is a common occurrence in hepatocellular carcinoma, with incidence ranging from 10% to 40% [4]. Its occurrence is associated with a poor prognosis in cases of hepatocellular carcinoma, especially when the thrombus involves the main portal vein [4]. It is also rarely seen in malignancies arising from the gastrointestinal tract, as a result of direct extension from either the primary tumor or hepatic metastasis. On the other hand, only a few cases of portal vein tumor thrombus arising from carcinoma of GB have been reported in the literature [2,3]. Zhang et al. reported a case of carcinoma of GB invading segment four of the liver, with direct extension into the right portal vein, resulting in a tumor thrombus [2]. Iyomasa et al. described a case of hepatic metastasis from carcinoma of GB invading the left portal vein [3]. Surgical resection was successfully done in both these cases, with extended right hepatectomy performed in the former and extended left hepatectomy performed in the latter case [2,3]. In our case, however, extensive encasement of the main portal vein and the common hepatic artery rendered the tumor unresectable. In addition to portal vein invasion, an unusual case of tumor thrombus in the superior vena cava has also been reported [5].

It has been shown in prior studies that the microscopic portal tract involvement in GB carcinoma is an important mode of tumor spread into the liver [6]. In a retrospective study of 42 patients by Wakai et al., although 24 patients were shown to have microscopic portal tract invasion, venous invasion was seen in only two patients [6]. The aggressive nature of the tumor in our case as evidenced by intravascular tumor extension and rapid-onset obstructive jaundice is likely because of the tumor being of adenosquamous type. It has been shown that adenosquamous carcinoma of GB presents at a more advanced stage of the disease and has lower overall survival rates than the more common adenocarcinoma variety [7]. The high propensity for invasion is because of the squamous component, which has a high proliferation rate [8]. One of the few cases of portal vein tumor thrombus in GB carcinoma has been reported in adenosquamous carcinoma [3].

## Conclusions

Adenosquamous carcinoma is an aggressive type of GB carcinoma with rapid disease progression and poor prognosis. It can rarely cause portal vein tumor thrombus, and its early identification is crucial for the initiation of appropriate treatment.
